# Prognostic value of aberrantly expressed methylation genes in human hepatocellular carcinoma

**DOI:** 10.1042/BSR20192593

**Published:** 2020-10-05

**Authors:** Limin Zhen, Gang Ning, Lina Wu, Yongyuan Zheng, Fangji Yang, Tongtong Chen, Wenxiong Xu, Ying Liu, Chan Xie, Liang Peng

**Affiliations:** 1 Department of Infectious Diseases, Third Affiliated Hospital of Sun Yat-Sen University, Guangzhou 510630, China; 2 Department of Gastroenterology and Hepatology, Guangzhou Digestive Diseases Center, Guangzhou First People's Hospital, South China University of Technology, Guangzhou, Guangdong Province 510180,China

**Keywords:** CDC5L, hepatocellular carcinoma, MERTK, methylation, prognosis, RHOA

## Abstract

Objectives: To identify the prognostic value of aberrantly methylated differentially expressed genes (DEGs) in hepatocellular carcinoma (HCC) and to explore the underlying mechanisms of tumorigenesis.

Methods: Gene expression profiles (GSE65372 and GSE37988) were analyzed using GEO2R to obtain aberrantly methylated DEGs. Functional enrichment analysis of screened genes was performed by the Database for Annotation, Visualization, and Integrated Discovery (DAVID). Cytoscape software was used to analyze the PPI network and to select hub genes. Transcriptional and proteinic expression data of hub genes were obtained through UALCAN and the Human Protein Reference Database. Finally, we analyzed the prognostic value of hub genes with the Kaplan–Meier Plotter and MethSurv database.

Results: In total, 24 up-hypomethylated oncogenes and 37 down-hypermethylated tumor suppressor genes (TSGs) were identified, and 8 hub genes, including 4 up-hypomethylated oncogenes (CDC5L, MERTK, RHOA and YBX1) and 4 down-hypermethylated TSGs (BCR, DFFA, SCUBE2 and TP63), were selected by PPI. Higher expression of methylated CDC5L-cg05671347, MERTK-cg08279316, RHOA-cg05657651 and YBX1-cg16306148, and lower expression of methylated BCR-cg25410636, DFFA-cg20696875, SCUBE2-cg19000089 and TP63-cg06520450, were associated with better overall survival (OS) in HCC patients. Multivariate analysis also showed they were independent prognostic factors for OS of HCC patients.

Conclusions: In summary, different expression of methylated genes above mentioned were associated with better prognosis in HCC patients. Altering the methylation status of these genes may be a therapeutic target for HCC, but it should be further evaluated in clinical studies.

## Introduction

Hepatocellular carcinoma (HCC) is a global malignant disease, ranking third in cancer-related mortality and causing more than 600,000 deaths each year [1,2]. The mortality caused by HCC has increased significantly in the past 20 years, and the deaths in the Asia-Pacific region account for the vast majority in the world [3]. At present, the main treatments for HCC are surgical resection, liver transplantation, and interventional therapy. However, the long-term prognosis is unsatisfactory, and the 5-year survival rate is less than 30% [4]. A number of factors can cause HCC, such as chronic viral infections (hepatitis B virus and hepatitis C virus), the deposition of iron and copper, fat accumulation, and so on [5].

It has been found that the occurrence and development of HCC is a multistage process that is caused by the inactivation of tumor suppressor genes (TSGs) or the activation of proto-oncogenes by genetic alterations and epigenetic abnormalities. As an important part of epigenetic regulation, DNA methylation has been found to play a pivotal role in tumorigenesis [6,7].

In HCC, abnormal methylation can affect the expression and functions of hub genes, thus taking part in various processes of HCC development and progression [8]. Even though many studies have been performed to find aberrantly methylated genes in HCC [9–11], it is limited for individual studies in overlapping gene profiling and it may be not enough to find pivotal genes and mechanisms in HCC. Therefore, the integrated gene profiles and their relationship are still not addressed clearly. In the study, we combined gene expression levels and gene methylation profiles in HCC and explored to confirm the key aberrantly methylated genes and their relationship, thus helping to identify biomarkers for the diagnosis and prognosis of HCC.

## Materials and methods

### Gene Expression Omnibus (GEO), ONGene and TSGene database

GEO (https://www.ncbi.nlm.nih.gov/geo/) is an online microarray or gene profiling database that is developed by NCBI. We used GEO2R (http://www.ncbi.nlm.nih.gov/geo/geo2r/), an online analysis tool, to identify the differentially expressed genes (DEGs) and differentially methylated genes (DMGs). The expression profiling from GSE65372, subsuming 39 HCC tumors and 15 normal liver tissues, was obtained from GPL14951 Platforms. The gene methylated profiling was gained from GSE37988, which included 62 HCC tumors and 62 adjacent non-tumor tissues, based on GPL8490. The cut-off criteria of DEGs was *P*-value < 0.05, and | logFC | > 1. DMGs with *P*-value < 0.05 and logFC < 0 regarded as hypomethylation, and with *P*-value < 0.05 and logFC > 0 as hypermethylation. We downloaded the oncogene information from the ONGene database (http://ongene.bioinfo-minzhao.org/), and the TSG information from the TSGene database (https://bioinfo.uth.edu/TSGene/index.html). An online tool (http://bioinformatics.psb.ugent.be/webtools/Venn/) was used to overlap DEGs, DMGs, oncogenes and TSGs.

### Gene Ontology (GO) and pathway enrichment (Kyoto Encyclopedia of Genes and Genomes, KEGG) analysis

GO is a standardized functional category system including three aspects: biological processes (BP), cellular components (CC), and molecular functions (MF), which offers a standardized series of dynamically latest annotations and describes the features of genes and gene products in organisms [12]. KEGG is one of the databases commonly used in pathway research, including metabolism, genetic processing, environmental processing, cellular processes, biological systems, diseases, and drug development [13]. Functions of hypomethylated up-regulated oncogenes and hypermethylated down-regulated TSGs were analyzed by GO and KEGG in the online Database for Annotation, Visualization, and Integrated Discovery (DAVID) (https://david.ncifcrf.gov/summary.jsp).

### Protein–protein interaction (PPI) network generation and hub genes selection

In organisms, proteins do not exist independently, and their functions must be regulated and mediated by other proteins. The implementation of this regulation or mediation first requires binding or interaction between proteins. Therefore, we constructed a PPI network to reveal the further functions of proteins. The PPI network was set up by the Search Tool for the Retrieval of Interacting Genes (STRING) database (https://string-db.org/cgi/input.pl). Next, we used the Cytoscape software to analyze the network and to select hub genes by cytoHubba. We used Molecular Complex Detection (MCODE) by Cytoscape software to filter modules. Then hub genes were confirmed with an algorithm degree >10 [14].

### Transcriptional expression of hub genes in HCC

UALCAN (http://ualcan.path.uab.edu) is an interactive web resource for analyzing cancer gene expression data and clinical data of 31 cancer types from the TCGA database [15]. We used the database to analyze transcriptional expression of target genes of HCC between tumor and normal samples and the association of the transcriptional expression with relative clinicopathologic parameters.

### Protein expression of hub genes in HCC

The Human Protein Atlas (https://www.proteinatlas.org) is a website tool, which is dedicated to providing tissues and cell distribution information for 24,000 human proteins. This database uses proprietary antibodies to examine the distribution and expression of each protein in 48 normal human tissues, 20 tumor tissues, 47 cell lines, and 12 blood cells by immunohistochemistry (IHC) [16]. In the present study, we compared the protein expression of different genes between human normal and HCC tissues by IHC levels.

### Survival analysis of hub genes based on mRNA expression

The prognostic value of 8 hub genes expression levels was analyzed by a free online database, KaplannMeier (http://kmplot.com/analysis/), which was established using gene expression data and survival information of liver cancer and four other types of cancer including breast cancer, ovarian cancer, lung cancer, and gastric cancer [17–19]. Briefly, 8 different hub genes were input to the database (http://kmplot.com/analysis/index.php?p=service&cancer=liver_rnaseq). The median values of mRNA expression were used to differentiate high and low expression groupsin patients with cancer and were validated by K-M survival curves. The median values of mRNA expression, HRs, 95%CIs and p values were displayed by K-M plotter. A *P* value < 0.05 was considered statically significant.

### Survival analysis of hub genes methylation

The prognostic role of the methylation of 8 hub genes was analyzed using the MethSurv Database (https://biit.cs.ut.ee/methsurv/). It is an open web tool to evaluate the prognostic values of CpG methylation data. This database can provide the overall survival (OS) with DNA methylation levels in univariable and multivariable survival analysis. All the information was based on CpG methylation that includes 7358 methylomes from 25 kinds of human cancers, and the methylome data are from The Cancer Genome Atlas (TCGA) [20]. On the webpage, we can obtain much important information about single CpG analysis, region-based analysis, and so on [20]. We employed the MethSurv Database to screen the different methylated sites of 8 hub genes. Then, we validated the most pivotal methylated site associated with HCC patient outcomes.

## Results

### Identification of DEGs and DMGs in patients with HCC

**Figure 1:**
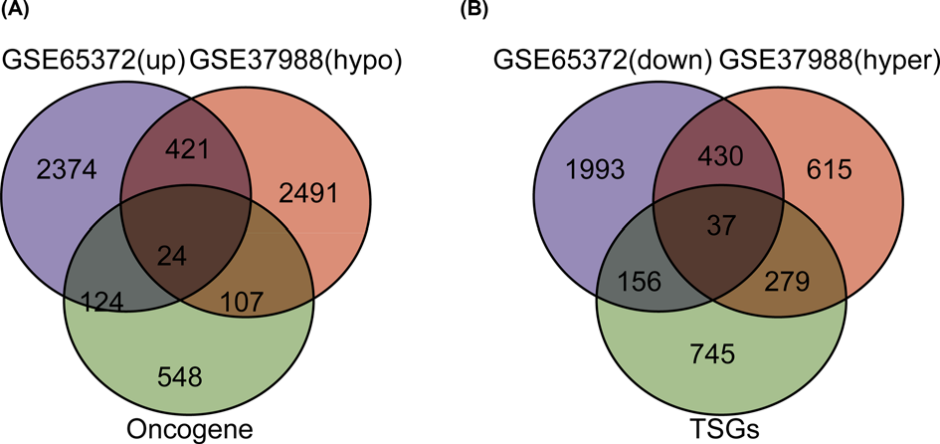
Identification of aberrantly methylated and differentially expressed genes, overlapping with oncogenes and tumor suppressor genes (TSGs) (**A**) Four hundred and forty-five hypomethylated up-regulated genes were identified, and twenty-four of them were oncogenes. (**B**) Four hundred and sixty-seven hypermethylated and down-regulated genes were identified, and thirty-seven of them were TSGs.

We obtained DEGs from GSE65372 and DMGs from GSE37988. Overlapping the up-regulated genes, hypomethylated genes and oncogenes, we obtained 445 hypomethylated and up-regulated genes, and 24 of them were oncogenes (Figure 1A). Similarly, 467 hypermethylated and down-regulated genes were obtained, and 37 of them were TSGs (Figure 1B).

### Further functions and pathways of hypomethylated up-regulated oncogenes and hypermethylated down-regulated TSGs in HCC patients

To explore the further functions and pathways, DAVID was performed to analyze the information of hypomethylated up-regulated oncogenes and hypermethylated down-regulated TSGs, respectively. A *P* value < 0.05 was regarded as significant. As shown in Table 1, hypomethylated up-regulated oncogenes were enriched in the negative regulation of the apoptotic process, cell proliferation, morphogenesis of an epithelial fold, positive regulation of NF-kappaB transcription factor activity, positive regulation of I-kappaB kinase/NF-kappaB signaling, which played an important role in biological procession. For hypermethylated down-regulated TSGs, biological processes, such as response to gamma radiation, odontogenesis of dentin-containing tooth, positive regulation of the apoptotic process, and negative regulation of cell proliferation, were significantly enriched after GO annotation. The results of KEGG pathway enrichment were shown in Table 2. The enrichment analysis suggested that pathways in cancer, sphingolipid signaling, platelet activation, regulation of actin cytoskeleton, and the rap1 signaling were in hypomethylated up-regulated oncogenes, while the neurotrophin signaling pathway, apoptosis, and pathways in cancer were in hypermethylated down-regulated TSGs.

**Table 1:** Gene ontology analysis of aberrantly methylated-differentially expressed oncogenes and TSGs in hepatocellular carcinoma

Category	GO analysis	Term	Gene count	%	*P* value
Up-regulated and hypomethylated expression					
	GOTERM_BP_DIRECT	GO:0043066∼negative regulation of apoptotic process	5	7.91	3.11E-04
	GOTERM_BP_DIRECT	GO:0008283∼cell proliferation	4	6.33	1.31E-03
	GOTERM_BP_DIRECT	GO:0060571∼morphogenesis of an epithelial fold	2	3.16	4.55E-03
	GOTERM_BP_DIRECT	GO:0051092∼positive regulation of NF-kappaB transcription factor activity	3	4.74	8.30E-03
	GOTERM_BP_DIRECT	GO:0043123∼positive regulation of I-kappaB kinase/NF-kappaB signaling	3	4.74	1.57E-02
	GOTERM_CC_DIRECT	GO:0005925∼focal adhesion	5	7.91	6.56E-04
	GOTERM_CC_DIRECT	GO:0005730∼nucleolus	6	9.49	8.58E-04
	GOTERM_CC_DIRECT	GO:0030496∼midbody	3	4.74	7.44E-3
	GOTERM_CC_DIRECT	GO:0070062∼extracellular exosome	8	12.65	1.52E-02
	GOTERM_CC_DIRECT	GO:0043234∼protein complex	3	4.74	2.26E-02
	GOTERM_MF_DIRECT	GO:0001077∼transcriptional activator activity, RNA polymerase II core promoter proximal region sequence-specific binding	4	6.33	3.15E-03
	GOTERM_MF_DIRECT	GO:0000978∼RNA polymerase II core promoter proximal region sequence-specific DNA binding	4	6.33	9.03E-03
	GOTERM_MF_DIRECT	GO:0004871∼signal transducer activity	3	4.74	1.87E-02
	GOTERM_MF_DIRECT	GO:0031683∼G-protein beta/gamma-subunit complex binding	2	3.16	2.38E-02
	GOTERM_MF_DIRECT	GO:0003924∼GTPase activity	3	4.74	3.12E-02
Down-regulated and hypermethylated expression					
	GOTERM_BP_DIRECT	GO:0010332∼response to gamma radiation	4	7.04	1.76E-05
	GOTERM_BP_DIRECT	GO:0042475∼odontogenesis of dentin-containing tooth	4	7.04	8.69E-05
	GOTERM_BP_DIRECT	GO:0043065∼positive regulation of apoptotic process	5	8.80	1.17E-04
	GOTERM_BP_DIRECT	GO:0008285∼negative regulation of cell proliferation	5	8.80	1.17E-03
	GOTERM_BP_DIRECT	GO:0000122∼negative regulation of transcription from RNA polymerase II promoter	6	10.56	1.27E-03
	GOTERM_CC_DIRECT	GO:0005829∼cytosol	12	21.12	5.66E-07
	GOTERM_CC_DIRECT	GO:0016363∼nuclear matrix	4	7.04	1.41E-04
	GOTERM_CC_DIRECT	GO:0000790∼nuclear chromatin	4	7.04	2.67E-03
	GOTERM_CC_DIRECT	GO:0035097∼histone methyltransferase complex	2	3.52	1.25E-02
	GOTERM_CC_DIRECT	GO:0000159∼protein phosphatase type 2A complex	2	3.52	3.70E-02
	GOTERM_MF_DIRECT	GO:0008601∼protein phosphatase type 2A regulator activity	2	3.52	2.89E-02

**Table 2:** KEGG pathway analysis of aberrantly methylated-differentially expressed oncogenes and TSGs in hepatocellular carcinoma

Pathway ID	Pathway name	Gene no.	%	*P* value	Genes
Up-regulated and hypomethylated expression					
hsa05200:	Pathways in cancer	6	9.50	3.59E-04	GNA13, EGFR, AR, GNAI2, RHOA, PIK3R1
hsa04071	Sphingolipid signaling pathway	4	6.30	9.97E-04	GNA13, GNAI2, RHOA, PIK3R1
hsa04611	Platelet activation	4	6.30	1.18E-03	GNA13, GNAI2, RHOA, PIK3R1
hsa04810	Regulation of actin cytoskeleton	4	6.30	5.07E-03	GNA13, EGFR, RHOA, PIK3R1
hsa04015	Rap1 signaling pathway	4	6.30	5.20E-03	EGFR, GNAI2, RHOA, PIK3R1
Down-regulated and hypermethylated expression					
hsa04722	Neurotrophin signaling pathway	4	7.04	5.53E-03	PRDM4, BAX, FOXO3, PRKCD
hsa04210	Apoptosis	3	5.28	1.38E-02	DFFA, BAX, CASP8
hsa05200	Pathways in cancer	5	8.80	2.94E-02	BMP4, BCR, BAX, CASP8, PML

### Protein–protein interaction (PPI) network analysis

**Figure 2:**
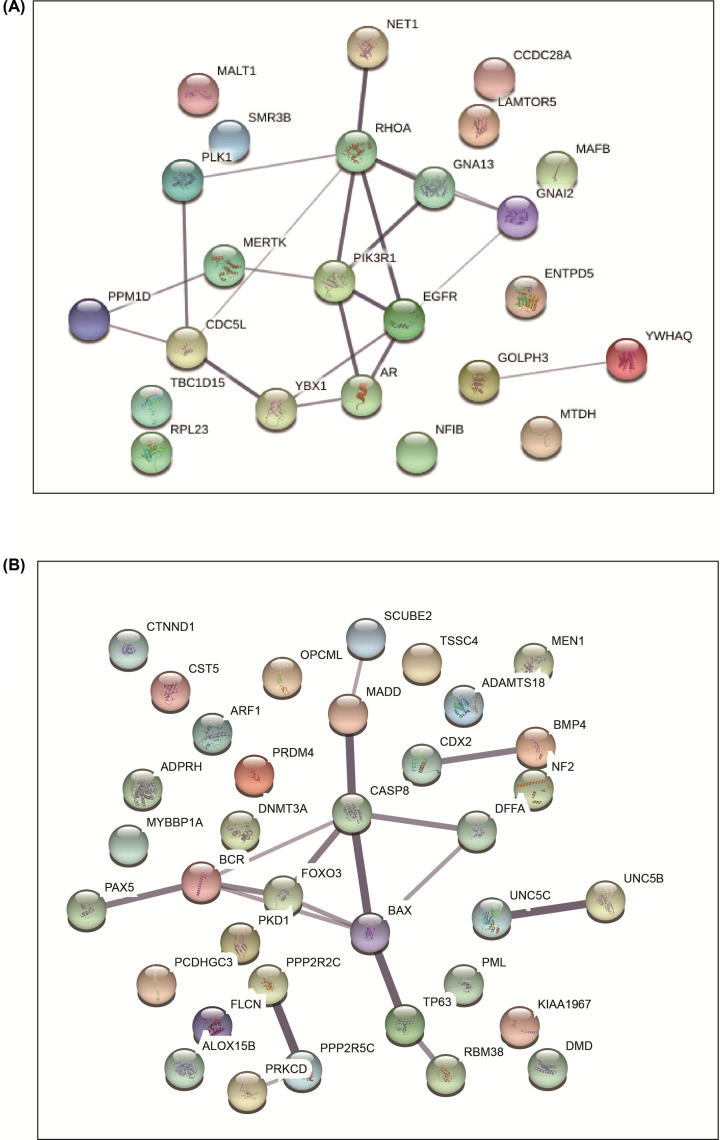
The PPI network for aberrantly methylated differentially expressed genes (**A**) PPI for hypomethylated up-regulated oncogenes. (**B**) PPI for hypermethylated down-regulated TSGs.

The PPI network was constructed by STRING database. There were 24 nodes and 21 edges for the 24 hypomethylated up-regulated oncogenes, and the PPI enrichment *P* value was 0.029 (Figure 2A). For 37 hypermethylated down-regulated TSGs, 36 nodes and 17 edges were identified with an enrichment *P* value of 0.000669 (Figure 2B). Next, we analyzed the PPI network data by Cytoscape software using the cytoHubba program to select the hub genes. In total, we identified 8 hub genes, 4 hypomethylated up-regulated oncogenes (CDC5L, MERTK, RHOA and YBX1) and 4 hypermethylated down-regulated TSGs (BCR, DFFA, SCUBE2, and TP63).

### The transcriptional and proteinic expression levels of 8 hub genes in HCC patients

**Figure 3:**
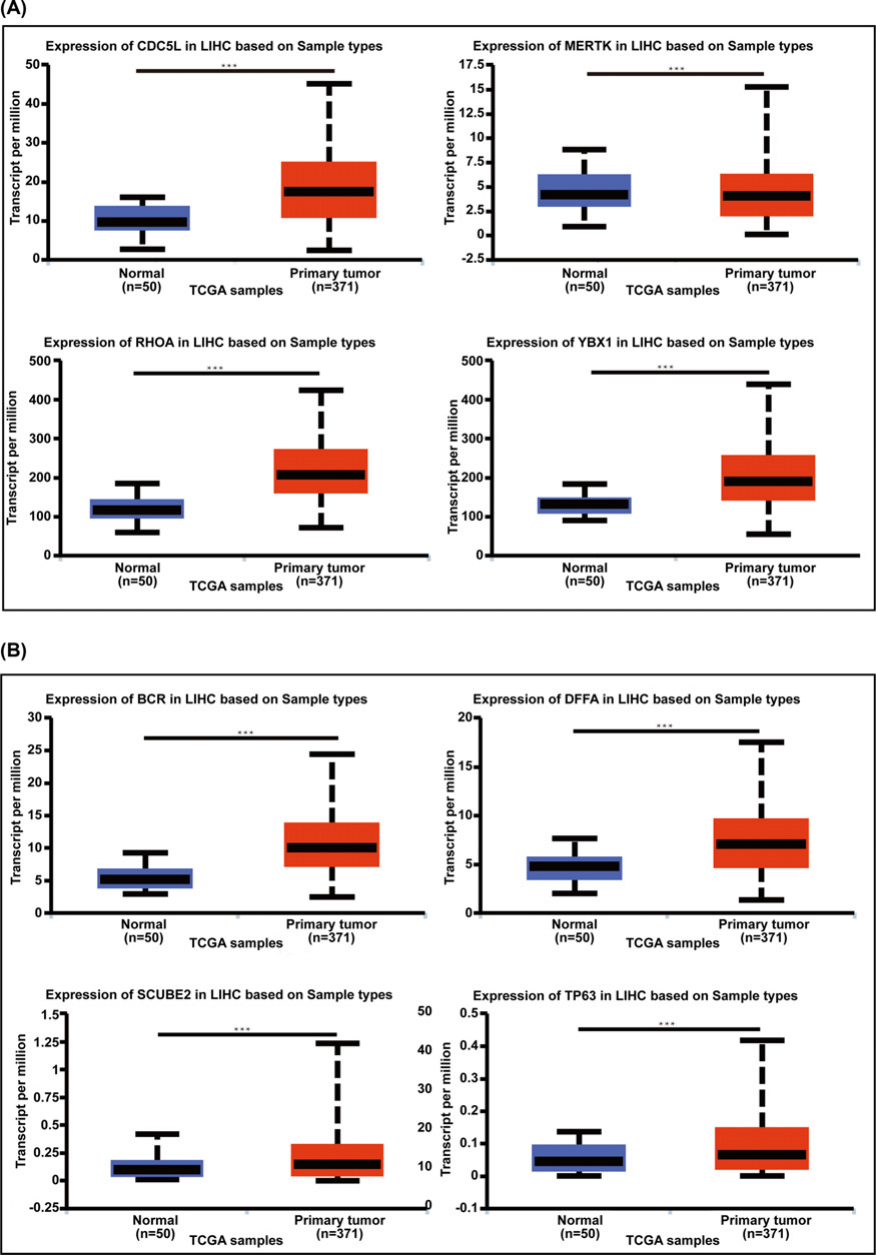
Measuring mRNA expression of 8 hub genes in the UALCAN (**A**) Box plots of hypomethylated up-regulated oncogenes (CDC5L, MERTK, RHOA, YBX1) in primary HCC tissues and normal liver samples. (**B**) Box plots of hypermethylated down-regulated TSGs (BCR, DFFA, SCUBE2, TP63) in primary HCC tissues and normal liver samples; ***P<0.001.

**Figure 4:**
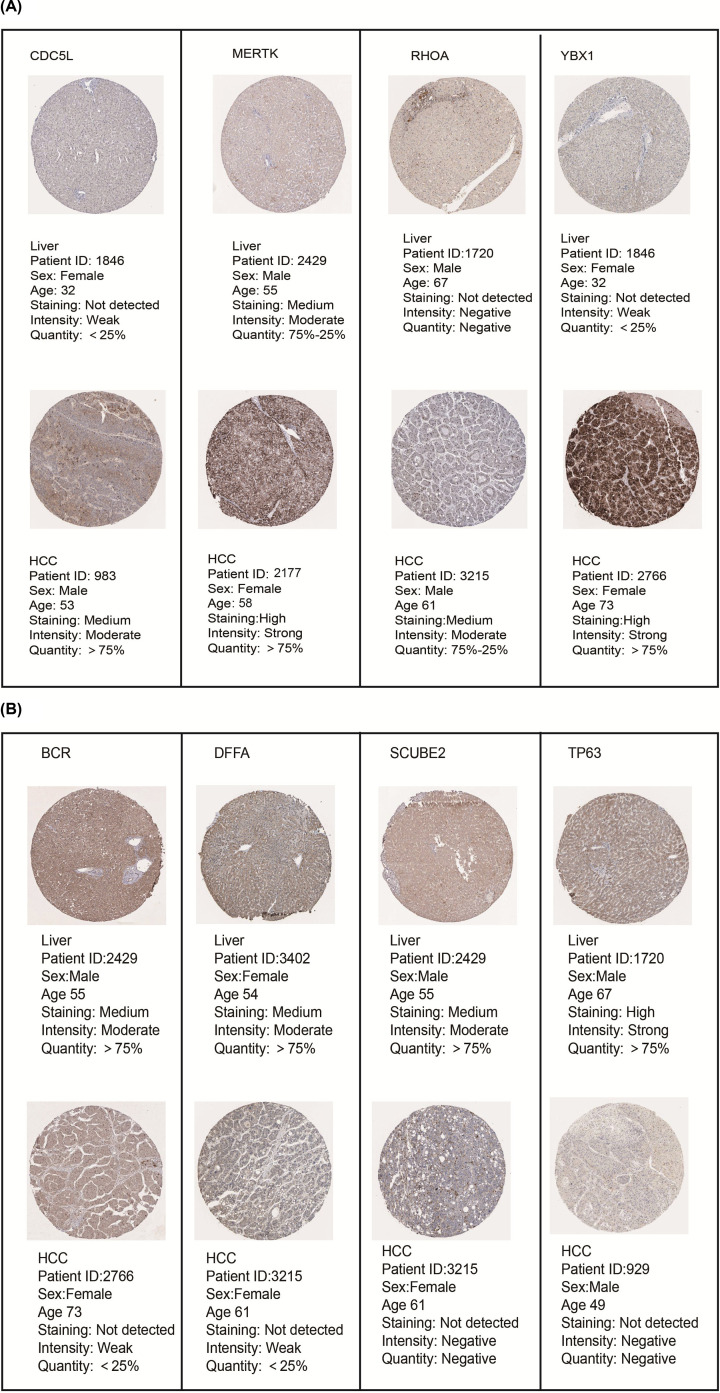
Relative immunohistochemistry results of 8 hub genes in HCC tissues and normal liver tissues from Human Protein Atlas database (**A**) The protein expression levels of hypomethylated up-regulated oncogenes in HCC and normal liver tissues by IHC images. (**B**) The protein expression levels of hypermethylated down-regulated TSGs in HCC and normal liver tissues.

The mRNA expression levels of 8 hub genes were evaluated in the UALCAN database, which involves 31 cancer types of TCGA by 3 RNA-seq and clinical datasets. As shown in Figure 3A, mRNA expression of CDC5L (*P*<1.00E-12), MERTK (*P*=3.77E-05), RHOA (*P*=1.62E-12), and YBX1 (*P*=1.62E-12) were obviously higher in HCC tissues compared with normal liver samples. In contrast, mRNA levels of BCR (*P*<1.00E-12), DFFA (*P*=1.62E-12), SCUBE2 (*P*=7.73E-05) and TP63 (*P*=4.42E-04) were found to be significantly lower in HCC compared with normal liver tissues (Figure 3B). To further explore the protein expression level of 8 hub genes, we used the Human Protein Atlas database. Medium expression of CDC5L and RHOA, high expression of MERTK and YBX1 was found in HCC tissues by IHC staining, while medium expression of MERTK was observed; CDC5L, RHOA, and YBX1 were not detected in normal liver tissues (Figure 4A). As shown in Figure 4B, protein expression was not detected in HCC patient tissues for any hypermethylated down-regulated TSGs, while medium expression of BCR, DFFA and SCUBE2, high expression of TP63 was found in normal liver samples. Taken together, our results showed that higher transcriptional and proteinic expression of CDC5L, MERTK, RHOA and YBX1, and lower transcriptional and proteinic expression of BCR, DFFA, SCUBE2 and TP63, were observed in patients with HCC.

### Prognostic value of mRNA expression and DNA methylation expression of 8 hub genes in HCC PATIENTS

**Figure 5:**
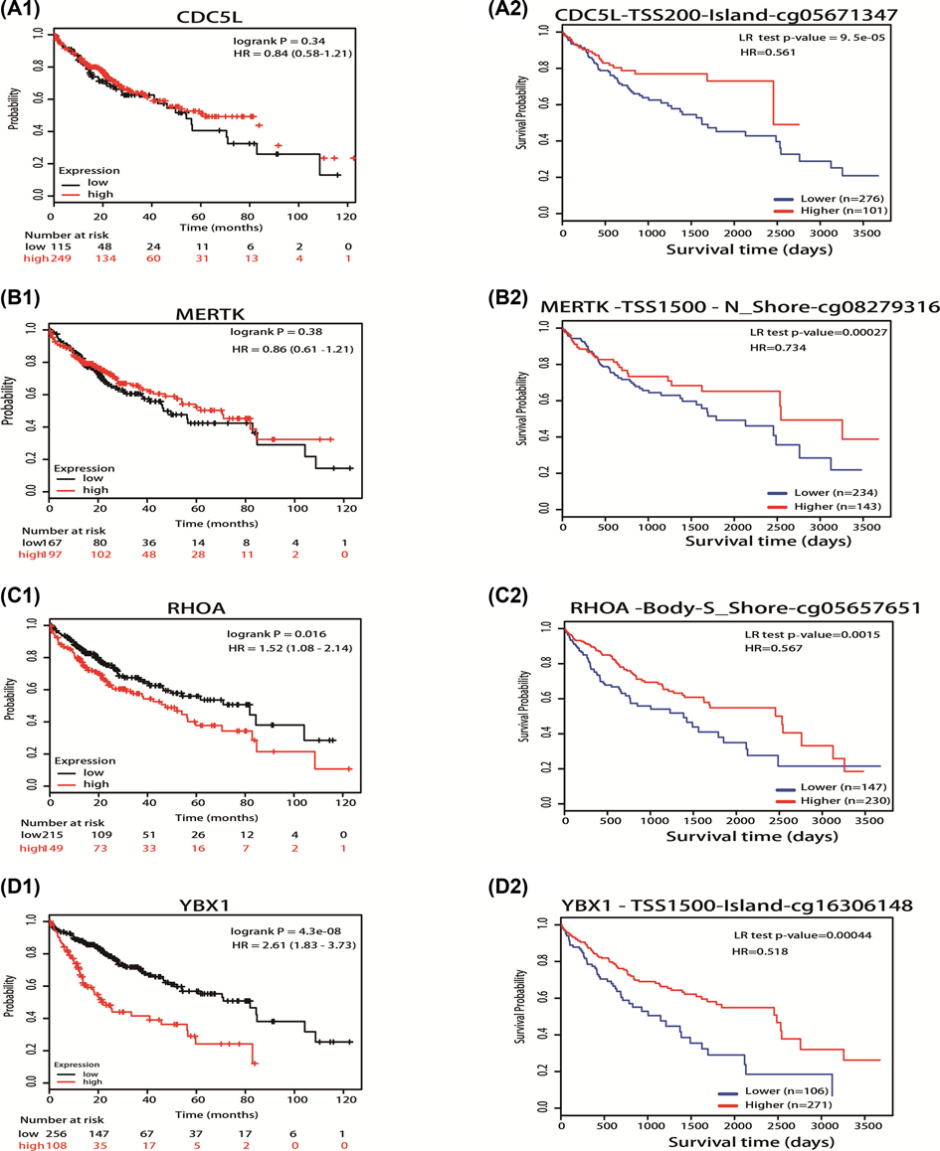
Prognostic value of mRNA expression (Kaplan-Meier plotter), methylation of hypomethylated upregulated oncogenes in HCC patients (MethSurv) Figure 5 (**A1**), (**B1**), (**C1**), (**D1**) showed the relation of mRNA expression of hypomethylated upregulated oncogenes with the prognosis in HCC patients using Kaplan–Meier plotter (http://kmplot.com/analysis/). Figure 5 (**A2**), (**B2**), (**C2**), (**D2**) showed the results of methylated level of hypomethylated up-regulated oncogenes with the prognosis in HCC patients by univariate analysis using MethSurv (https://biit.cs.ut.ee/methsurv/).

To evaluate the relationship of mRNA expression and DNA methylation expression of 8 hub genes with survival in liver cancer patients, we used two databases, including Kaplan–Meier plotter (http://kmplot.com/analysis/) and MethSurv (https://biit.cs.ut.ee/methsurv/). As is shown in Figure 5, HCC patients with lower mRNA levels of RHOA (HR = 1.52, 95% CI: 1.08–2.14, *P*=0.016) and YBX1 (HR = 2.61, 95% CI: 1.83–3.73, *P*=4.3E-08) had higher overall survival (OS) (Figure 5C1,D1), while mRNA expression of CDC5L (HR = 0.84, 95% CI: 0.58–1.21, *P*=0.34) and MERTK (HR = 0.86, 95% CI: 0.61–1.21, *P*=0.38) was not associated with liver cancer patient survival (Figure 5A1,B1). In addition, we also used the MethSurv database to analyze the prognostic value of the DNA methylation of these genes. Our results showed that univariable and multivariable survival analyses according to the methylation of CpG sites by Cox proportional-hazards models based on TCGA database (level 3 data, HM450K). In the univariable analysis, higher DNA methylation of these four hypomethylated up-regulated oncogenes was associated with significantly higher OS in HCC patients (CDC5L-cg05671347, HR = 0.466, 95% CI: 0.298–0.727, *P*=9.5E-05; MERTK-cg08279316, HR = 0.671, 95% CI: 0.463–0.971, *P*=0.00027; RHOA-cg05657651, HR = 0.567, 95% CI: 0.402–0.801, *P*=0.0015; YBX1-cg16306148, HR = 0.518, 95% CI: 0.364–0.739, *P*=0.00044) (Figure 5A2,B2,C2,D2).

**Figure 6:**
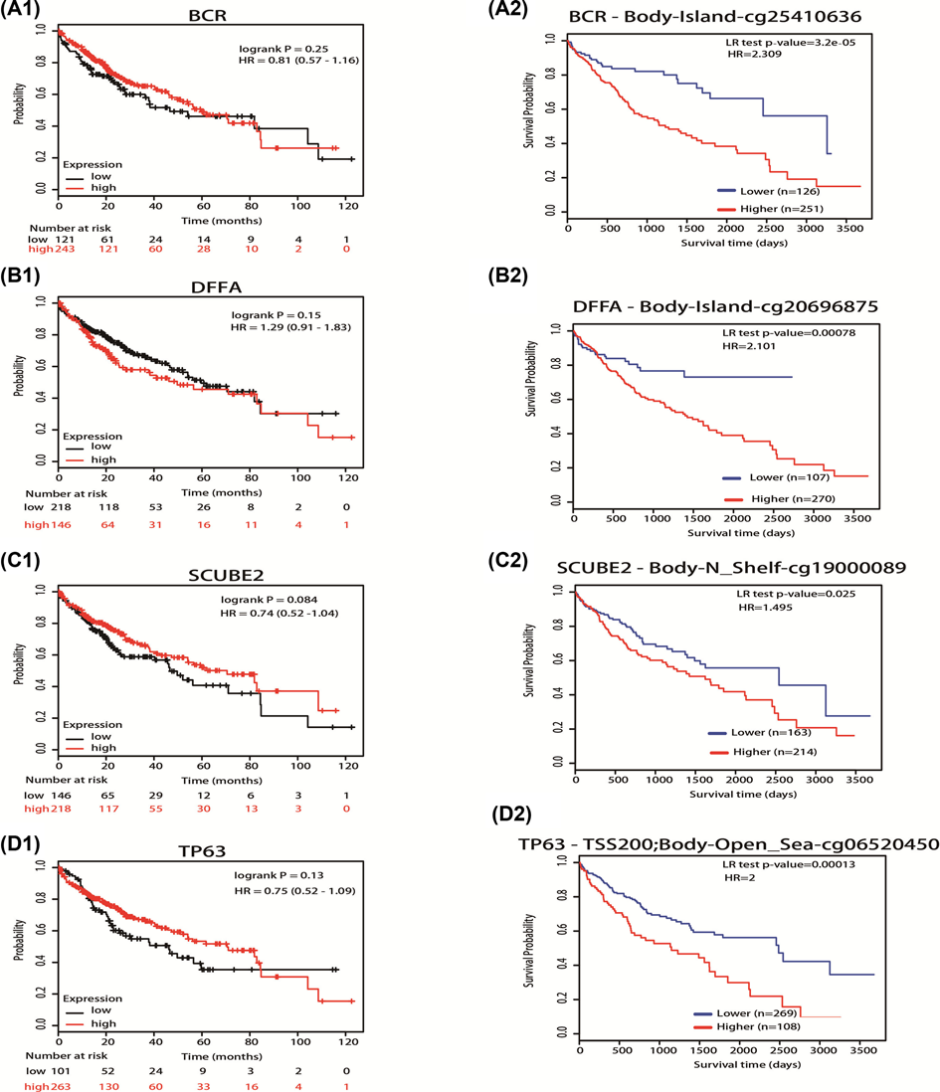
Prognostic value of mRNA expression, methylation of hypermethylated downregulated TSGs in HCC patients Figure 6 (**A1**), (**B1**), (**C1**), (**D1**) showed the correlation of mRNA expression of hypermethylated down-regulated TSGs with the prognosis in HCC patients using Kaplan–Meier plotter. Figure 6 (**A2**), (**B2**), (**C2**), (**D2**) showed the results of methylated level of hypermethylated down-regulated TSGs with the prognosis in HCC patients by univariate analysis using MethSurv (https://biit.cs.ut.ee/methsurv/).

Next, we also used Kaplan–Meier plotter to analyze the relationship of mRNA expression of hypermethylated down-regulated TSGs with OS in HCC patients. We found that mRNA expression levels of all the hypermethylated down-regulated TSGs (BCR, DFFA, SCUBE2, TP63) were not associated with OS (BCR, HR = 0.81, 95% CI: 0.57–1.16, *P*=0.25; DFFA, HR = 1.29, 95% CI: 0.91–1.83, *P*=0.15; SCUBE2, HR = 0.74, 95% CI: 0.52–1.04, *P*=0.084; TP63, HR = 0.75, 95% CI: 0.52–1.09, *P*=0.13) (Figure 6A1,B1,C1,D1). However, the methylation of these 4 genes was markedly correlative with HCC patient survival. Our results revealed that HCC patients with hypermethylation of these four genes had better OS (BCR-cg25410636, HR = 2.309, 95% CI: 1.51–3.529, *P*=3.2E-05; DFFA-cg20696875, HR = 2.102, 95% CI: 1.315–3.357, *P*=0.00078; SCUBE2-cg19000089, HR = 1.495, 95% CI: 1.048–2.134, *P*=0.025; TP63-cg06520450, HR = 2.00, 95% CI: 1.415–2.826, *P*=0.00013) (Figure 6A2,B2,C2,D2). These results indicated that DNA methylation levels of these hub genes were significantly associated with the prognosis of liver cancer patients and that they may be exploited as useful biomarkers for the prediction of liver cancer patient survival.

Finally, we then tried to assess the independent prognostic value of DNA methylation levels of these hub genes in terms of OS in liver cancer patients. In multivariable survival analysis, clinical information including age, sex, clinical stage, grade, weight and height were adjusted. For hypomethylated up-regulated oncogenes, our results showed that HCC patients with higher methylated expression of CDC5L-cg05671347 (HR = 0.561, 95% CI: 0.328–959, *P*=0.035), RHOA-cg05657651 (HR = 0.567, 95% CI: 0.402–0.801, *P*=0.0013), and YBX1-cg16304148 (HR = 0.518, 95% CI: 0.364–739, *P*=0.00028) had higher OS. However, the methylation level of MERTK-cg08279316 was not associated with OS in HCC (HR = 0.734, 95% CI: 0.479–1.124, *P*=0.15) (Table 3). In regard to hypermethylated down-regulated TSGs, methylation of four genes was significantly related with OS in HCC patients. Our results showed that lower methylated expression of these four genes was associated with better OS (BCR-cg25410636, HR = 1.759, 95% CI: 1.101–2.81, *P*=0.018; DFFA-cg20696875, HR = 2.124, 95% CI: 1.26–3.58, *P*=0.0047; SCUBE2-cg19000089, HR = 1.631, 95% CI: 1.083–2.455, *P*=0.019; TP63-cg06520450, HR = 1.991, 95% CI: 1.324–2.994, *P*=0.00094) (Table 3). Taken together, our results show that DNA methylation expressions of CDC5L-cg05671347, RHOA-cg05657651, YBX1-cg1630414, BCR-cg25410636, DFFA-cg20696875, SCUBE2-cg19000089 and TP63-cg06520450 were independent prognostic factors for OS in HCC patients.

**Table 3:** Survival analysis summary after covariate adjustment (including age, sex, stage, and grade) of aberrantly methylated-differentially expressed oncogenes and TSGs based on TCGA database in HCC patients

Gene symbol	HR	95% CI	Wald *P* value	Current split	Mean/q25/ maxstat	Range
Up-regulated and hypomethylated expression						
CDC5L-cg05671347 (hypermethylation)	0.561	0.328–0.959	0.035	Mean	0.06	0.021–0.314
MERTK-cg08279316 (hypermethylation)	0.734	0.479–1.124	0.15	Mean	0.03	0.018–0.239
RHOA-cg05657651 (hypermethylation)	0.567	0.402–0.801	0.0013	maxstat	0.919	0.484–0.963
YBX1-cg16306148 (hypermethylation)	0.518	0.364–0.739	0.00028	q25	0.017	0.013–0.052
Down-regulated and hypermethylated expression						
BCR-cg25410636 (hypermethylation)	1.759	1.101–2.81	0.018	maxstat	0.761	0.575–0.915
DFFA-cg20696875 (hypermethylation)	2.124	1.26–3.58	0.0047	maxstat	0.04	0.021–0.498
SCUBE2-cg19000089 (hypermethylation)	1.631	1.083–2.455	0.019	maxstat	0.766	0.074–0.932
TP63-cg06520450 (hypermethylation)	1.991	1.324–2.994	0.00094	maxstat	0.901	0.089–0.944

## Discussion

HCC is a multistage process caused by the inactivation of TSG or the activation of proto-oncogenes by genetic alterations and epigenetic abnormalities. Currently, it is found that hypermethylation of the CpG island in the promoter region of TSGs can enhance the spatial structure of chromatin, block the transcription of genes, silence the expression of TSGs, and down-regulate the expression of proto-oncogene by recruiting methylation-binding proteins and related complexes [21]. Therefore, the hypermethylation of the promoter region of the TSGs and the hypomethylation of the oncogene promoter region are closely related to oncogenesis of HCC. In our study, gene expression profiles and gene methylation profiles of HCC were jointly analyzed to find key aberrantly methylated genes, which may help to identify biomarker for diagnosis and prognosis of HCC.

It would greatly benefit the diagnosis, therapy and prognosis of HCC to illuminate the potential mechanisms of the initiation and evolution. In the present study, we identified 24 hypomethylated up-regulated oncogenes and 37 hypermethylated down-regulated TSGs by several kinds of bioinformatics online tools. Elucidating these genes in pathways and verifying hub genes with abnormal methylation may give us a new thinking for latent mechanisms of HCC. As was shown by GO analysis, hypomethylated oncogenes in HCC were enriched in biological processes, including the negative regulation of the apoptotic process and cell proliferation, while positive regulation of the apoptotic process, negative regulation of cell proliferation and negative regulation of transcription from RNA polymerase II promoter were enriched in hypermethylated TSGs. The molecular function of hypomethylated oncogenes was enriched with transcriptional activator activity, RNA polymerase II core promoter proximal region sequence-specific binding, and RNA polymerase II core promoter proximal region sequence-specific DNA binding. It is reasonable that minimal cell apoptosis, frequent cell proliferation, transcriptional activation of oncogenes and DNA-binding regulation are important in the progression of cancers, including HCC. KEGG pathway enrichment demonstrated that pathways in cancer were significantly enriched both in oncogenes and TSGs. These pathways include p53 signaling and Wnt, which are frequently dysregulated in HCC [22,23].

The free online tool, STRING, was used to constructed the PPI network; then, we screened eight hub genes by Cytoscape software, including four hypomethylated up-regulated oncogenes (CDC5L, MERTK, RHOA and YBX1) and four hypermethylated down-regulated TSGs (BCR, DFFA, SCUBE2, and TP63). Higher transcriptional and proteinic expression levels of CDC5L, MERTK, RHOA and YBX1, and lower transcriptional and proteinic expression levels of BCR, DFFA, SCUBE2 and TP63, were found in HCC patients. In addition, higher methylation of CDC5L-cg05671347, MERTK-cg08279316, RHOA-cg05657651 and YBX1-cg16306148, and lower methylation of BCR-cg25410636, DFFA-cg20696875, SCUBE2-cg19000089 and TP63-cg06520450, were significantly associated with better OS in HCC patients. Multivariate analysis also showed that higher methylation of CDC5L-cg05671347, RHOA-cg05657651 and YBX1-cg16306148, and lower methylation of BCR-cg25410636, DFFA-cg20696875, SCUBE2-cg19000089 and TP63-cg06520450, were independent prognostic factors for OS in HCC patients.

CDC5L is a DNA-binding protein and transcriptional activator involved in cell cycle control [24,25]. It has found that CDC5L is closely associated with cell division and cell proliferation. Studies have found that CDC5L was highly expressed in HCC tissues and was significantly related to AJCC stage, tumor size, and Ki-67. High expression of CDC5L was an independent prognostic factor for poor survival of HCC patients. *In vitro* studies showed that overexpression of CDC5L contributed to cell cycle progress of HCC cells, while down-regulation of CDC5L resulted in cell cycle arrest at G2/M phase and reduced cell proliferation of HCC cells [26]. Moreover, higher phosphorylation of CDC5L was found in HCC cell line, MHCC97-H (high metastasis), indicating that it may participate in the metastasis of HCC [27]. Similarly, in our study, higher transcriptional and proteinic expression levels of CDC5L were found in HCC patients. Additionally, higher methylation of CDC5L-cg05671347 was associated with better OS of HCC patients and was also an independent prognostic factor for OS of HCC patients. Together with other studies, our results suggest that CDC5L plays an important role in the tumorigenesis of HCC, and thus, is a potential prognostic biomarker for HCC patients.

MERTK is a member of the MER / AXL / TYRO3 receptor kinase family that transduces signals from the extracellular matrix to the cytoplasm by binding to several ligands, including LGALS3, TUB, TULP1 and GAS6. MERTK regulates many physiological processes, such as cell proliferation, migration, differentiation, and phagocytosis of apoptotic cells [28]. MERTK is overexpressed in a variety of tumors, and its overexpression promotes tumor cell proliferation, migration, and invasion [29–32]. Our results showed that the mRNA and protein expression levels of MERTK were higher in HCC tissues, and patients with lower methylation of MERTK-cg08279316 had a poorer prognosis in univariate analysis. However, through multivariate analysis, we found higher methylation of MERTK-cg08279316 was not associated with OS in HCC patients, which suggests that MERTK-cg08279316 may not be a good prognostic biomarker for HCC.

RHOA encodes a member of the Rho family of small GTPases that acts as a molecular switch in the signal transduction cascade [33]. Overexpression of this gene is associated with tumor cell proliferation and metastasis [34,35]. Gou et al found that high expression of RhoA protein was recognized in HCC compared with the paired nontumor tissues and was associated with poorer disease-free survival (DFS) in HCC patients, suggesting RhoA was a useful marker for predicting early recurrence in early-stage HCC [36]. Mechanistically, Fukui et al showed that down-regulation of RhoA expression led to a significant inhibition of cell growth, induction of apoptosis, and reduction in the migration of HepG2 and Hep3B cells [37]. Moreover, Galectin 3, MENA, long noncoding RNA AFAP1-AS1 could promote HCC cell proliferation and invasion via the up-regulation of RhoA/Rac2 signaling [38–40]. In our study, higher transcriptional and proteinic expression levels of RHOA were found in HCC patients. Additionally, higher methylation of RHOA-cg05657651 was associated with better OS of HCC patients and was also an independent prognostic factor for OS of HCC patients. Together with other studies, our results indicated that RHOA may be exploited as a potential prognostic biomarker for HCC patients.

YBX1 encodes a highly conserved cold shock domain protein that acts as a DNA and RNA binding protein involved in many cellular processes, including transcriptional regulation and translation, premRNA splicing, DNA repair and mRNA packaging [41]. Abnormal expression of this gene is associated with cell proliferation in many cancers, which may be a prognostic marker for poor prognosis and resistance in certain cancers, including HCC [42,43]. Ali et al. found lncRNA affected PAN-cancer by activating the YBX1/ hnRNPK complex through regulating the FGF/FGFR, PI3K/AKT and MAPK pathways [44]. Zhang et al. showed that in human gastric cancer, YBX1 interacted with HOXC-AS3 and took part in HOXC-AS3-mediated gene transcriptional regulation in tumorigenesis [45]. There were some studies demonstrated that YBX1 could affect the RNA binding during oxidative stress to promote the tumorigenesis [46,47]. In our study, elevated transcriptional and proteinic expressions of YBX1 were found in HCC patients, and in K-M plotter, patients with higher expression of YBX1 had a significantly poorer prognosis. In contrast, hypermethylation at cg16306148 was a good predictor of prognosis for HCC, suggesting it can be a prognosis marker.

The function of the normal BCR gene product is not clear, but the BCR/ABL fusion protein has been researched deeply. The abnormal expression of BCR/ABL may lead to many diseases, including chronic granulocytes leukemia, acute lymphocyte leukemia and the related pathways, including endometrial cancer and the PI3K/Akt pathway [48–51]. However, there is limited research on the methylation status of the BCR gene in liver cancer. Miyazaki et al. used IHC and Western blot to show that the BCR protein level was higher in HCC than in liver tissues adjacent to HCC tissue [52]. In contrast, our results showed that the expression levels of BCR mRNA and protein in HCC tissues were decreased, but it was not an independent risk factor for the prognosis of HCC in K-M plotter. However, we found lower methylation of its cg25410636 site led to a better prognosis for HCC patients. BCR-cg25410636 may become a potential prognostic factor in HCC, and we need further research to confirm it.

DFFA is an apoptosis-related gene that is expressed in many normal tissues such as liver, colon, lung, breast, and epithelial tissues, and it is overexpressed in peripheral blood mononuclear cells. Its expression is suppressed to different degrees in tumor cells, including gastrointestinal cancer, bladder cancer, and so on [53,54]. Toraih et al observed that DFFA was the target gene of microRNA, through regulating cell apoptosis to affect the initiation and development of renal cell carcinoma [53]. Kekeeva et al. elucidated that DFFA was associated with bladder carcinogenesis [54]. Pei et al. further demonstrated that AT2R promotes the tumorigenesis of bladder cancer by downregulation of DFFA [55]. There are few studies on the methylation of DFFA. In our study, we showed that although DFFA had low transcription and protein expression levels in HCC tissues, it was not an independent factor affecting prognosis. The lower methylation at cg20696875, the better prognosis in patients with HCC. The mechanism may be due to the suppressing expression after methylation of the DFFA promoter region in HCC tissues, which cannot be precisely regulated during cell differentiation, so it can successfully pass through the G/S phase checkpoint in the cell cycle and enter the cell differentiation and proliferative phase. This cell selection advantage leads to infinite proliferation, and it eventually promotes the development of HCC.

Similarly, SCUBE2 is an important TSG. It has attracted attention because of its low expression in breast cancer tissues [56,57]. However, studies on its methylation have not been reported. Lin et al. found that SCUBE2 regulated TGF-β signaling and vascular endothelial growth factor (VEGF)/vascular endothelial growth factor receptor 2 (VEGFR2) binding and activity (affecting VEGF signaling pathways), to influence biological processes [58]. In our study, although the mRNA and protein expressions of SCUBE2 were higher in HCC patients, these differences were not associated with the prognosis of HCC. However, the lower methylation at the SCUBE2-cg19000089 site, the better prognosis for patients with HCC. It may affect biological processes such as angiogenesis and reduce the blood supply of tumors by regulating the above-mentioned signaling pathways.

As a sequence-specific DNA-binding transcriptional activator or suppressor, TP63 may have to combine with TP73/p73 to initiate p53/TP53-dependent apoptosis in response to genotoxic damage and the presence of activated oncogenes. It activates the Notch signaling pathway by induction of JAG1 and JAG2. Papakonstantinou found different levels of methylation of TP63 in different subtypes of chronic lymphocytic leukemia [59]. Childs et al. identified that the alteration of TP63-rs9854771 might be a new risk region in pancreatic cancer via a genome-wide association study from multiple centers [60]. In the present study, we found that both mRNA and protein expression levels of TP63 were up-regulated in HCC tissues, though it was not a dependent prognostic factor in OS of HCC. However, we verified that the lower methylation of the cg06520450 locus, the better HCC prognosis, suggesting that this methylated site is a good biomarker for the prognosis in HCC patients.

There were several limitations in our study. First, although higher methylation of CDC5L-cg05671347, MERTK-cg08279316, RHOA-cg05657651 and YBX1-cg16306148, and lower methylation of BCR-cg25410636, DFFA-cg20696875, SCUBE2-cg19000089 and TP63-cg06520450, were associated with better OS in HCC patients, all the data analyzed in our study were retrieved from online databases. Additional studies with larger sample sizes are required to validate our findings and to explore the clinical application of the methylated sites in the treatment of HCC. Second, we did not assess the potential diagnostic and therapeutic roles of these methylated sites in HCC, so future studies are needed to explore whether they could be exploited as diagnostic markers or as therapeutic targets. Finally, we did not explore the potential mechanisms of distinct methylated sites in HCC. In future studies, it is worth investigating the detailed mechanism between the distinct methylated sites and HCC prognosis.

## Conclusions

Our results suggest that higher methylation of CDC5L-cg05671347, MERTK-cg08279316, RHOA-cg05657651 and YBX1-cg16306148, and lower methylation of BCR-cg25410636, DFFA-cg20696875, SCUBE2-cg19000089 and TP63-cg06520450, were associated with better OS in HCC patients, with the exception of MERTK-cg08279316, they were independent prognostic factors for OS in HCC patients. Because gene methylation is reversible, recovering the normal methylation status of these genes may be a new direction for reversing and treating HCC.

## Data Availability

The data used to support the findings of this study are available from the corresponding author upon request.

## Abbreviations

DEG: differentially expressed gene
HCC: hepatocellular carcinoma
IHC: immunohistochemistry
PPI: protein–protein interaction
VEGF: vascular endothelial growth factor
